# Genetic and epigenetic correlates of co-occurring behavioral problems in early childhood

**DOI:** 10.21203/rs.3.rs-10764908/v1

**Published:** 2026-10-27

**Authors:** Yunpeng Wang, Mona Bekkhus, Stella Tsotsi, Rosa Mulder, Zoe Hart, Christian Page, Nikolai Olavi Czajkowski, Wanlin Liu, Espen Røysamb, Ragnhild Nes, Aurora Oftedal, Robert Lyle, Laurie Hannigan, Ole Andreassen, Vilte Baltramonaityte, Esther Walton, Charlotte Cecil

**Affiliations:** University of Oslo; PROMENTA Research Centre, Department of Psychology, University of Oslo; Erasmus MC; Norwegian Institute of Public Health; Department of Medical Genetics, Oslo University Hospital; Oslo University Hospital & Institute of Clinical Medicine, University of Oslo; University of Bath; Erasmus Medical Centre, University Medical Center Rotterdam

## Abstract

Co-occurring internalizing and externalizing behavioral problems in childhood are associated with greater symptom persistence and increased psychiatric burden than either in isolation, yet their molecular correlates in early development remain poorly characterized. We analyzed behavioral profiles at ages three and five years in the Norwegian Mother, Father and Child Cohort Study (MoBa), using a multi-layer and multi-cohorts design integrating genome-wide association analysis, polygenic score analysis, epigenome-wide association analysis of cord blood DNA methylation, and co-methylation network analysis. Behavioral profiles were based on previously identified latent classes representing internalizing, aggression, and co-occurring problems at each age. Across six genome-wide association analyses, covering these three behavioral profiles at ages three and five, we identified one genome-wide significant locus for co-occurring problems at age five, led by rs73161798 at chromosome 13q12 (*P* = 3.26 × 10^− 8^). SNP-based heritability estimates were modest (h^2^_SNP_ ≈ 0.03–0.07) but became more consistently detectable from age three to five. Polygenic score analyses provided the most consistent molecular signal, with broader associations at age five across psychiatric, cognitive, and neurodevelopmental domains. In contrast, single-site cord-blood DNA methylation associations were sparse, identifying one age-three cytosine-phosphate-guanine site (CpG) associated with co-occurring problems, annotated to *TNKS* (cg10976481; *P* = 9.03 × 10^− 8^), and three age-five CpGs, including one in the *TENM4* locus (cg18224993; *P* = 6.38 × 10^− 8^). These methylation associations were then examined in two independent cohorts, ALSPAC and GenR. Co-methylation network analysis identified age-five modules associated with co-occurring profiles, with nominal enrichment for pathways related to neurodevelopment, synaptic organization, neuronal signaling, and plasticity. These findings thus suggest that molecular correlates become more consistently detectable as behavioral phenotypes become more stable across early childhood from age three to five.

## Introduction

Childhood behavioral problems are common, developmentally heterogeneous, and clinically consequential ^1–3^. Internalizing difficulties, such as anxiety, withdrawal and emotional symptoms, and externalizing behaviors, such as oppositionality and physical or relational aggression, are often studied as distinct domains ^4^. In early childhood, however, these problems frequently co-occur within the same child ^5,6^. This co-occurrence is clinically important because children exhibiting both internalizing and externalizing symptoms tend to experience more persistent difficulties and greater later psychiatric burden than children with single-domain symptom profiles ^7–11^. Despite this, molecular studies of early behavioral problems have largely focused on individual symptom domains, leaving the genetic and epigenetic correlates of co-occurring behavioral profiles poorly understood.

A central unresolved question is whether co-occurring behavioral problems primarily reflect overlapping liabilities already implicated in internalizing and externalizing traits, or whether they also mark a more developmentally distinct profile ^12,13^. Twin and genomic studies indicate that childhood psychopathology is shaped by both shared and domain-specific genetic influences, and psychiatric genetic studies increasingly support broad pleiotropic liability across emotional, behavioral, cognitive, and neurodevelopmental traits ^14–20^. At the same time, person-centered developmental studies show that co-occurring profiles can be identified early in life and are associated with elevated risk later in development^21^. These observations support a framework in which co-occurring behavioral problems are not viewed simply as the additive combination of internalizing and externalizing behavior, but rather as a potentially informative early-life phenotype that may index broader developmental vulnerability.

The timing of assessment is also critical ^10,22^. Behavioral problems measured at in early childhood may partly reflect transient variation in early self-regulation, language development, temperament, and parent-reported adjustment ^23^. By mid-childhood, behavioral profiles may be more stable and more closely aligned with emerging cognitive, emotional, and social regulation. Molecular correlates in early childhood may be subtle, yet they can provide early insight into emerging biological pathways that become more structured as behavioral phenotypes stabilize. This developmental perspective is particularly relevant for co-occurring problems, which may index broader liability more clearly once symptom patterns become more stable and differentiated. Common genetic variation provides one route to characterizing this liability. Genome-wide association analysis can identify individual loci associated with early behavioral profiles ^20,24^, while single nucleotide polymorphism (SNP)-based heritability and polygenic score (PGS) analyses can test whether childhood behavioral profiles reflect broader genetic liabilities for psychiatric, cognitive, and neurodevelopmental traits. We therefore selected PGSs across psychiatric, cognitive, educational, and neurodevelopmental domains to examine whether early behavioral profiles show genetic continuity with traits and disorders that commonly emerge or become clinically salient later in development. However, individual common-variant effects for early childhood behavior are expected to be small, and genome-wide significant findings may be difficult to detect even in large cohorts. Polygenic approaches may therefore provide a more sensitive framework for evaluating whether early behavioral profiles are developmentally linked to later psychiatric and cognitive vulnerability.

DNA methylation offers a complementary molecular perspective. Cord-blood methylation is measured before the emergence of later childhood behavioral profiles and may capture prenatal biological variation related to genetic regulation, fetal development, maternal exposures, cell composition, and other early-life processes. However, methylation associations with behavioral phenotypes are often weak, context-dependent, and difficult to interpret at the level of single CpG sites. Network-level approaches, which model correlated patterns of CpG sites as interconnected modules rather than isolated loci, may therefore be useful for identifying coordinated methylation structure even when individual CpG associations are sparse. Such approaches can provide biological context, while still requiring cautious interpretation because cord blood is not brain tissue and methylation signatures do not by themselves establish causal mechanisms.

Here, we used the Norwegian Mother, Father and Child Cohort Study (MoBa) ^25,26^, to characterize molecular correlates of early childhood internalizing, externalizing (indexed by aggression), and co-occurring behavioral profiles at ages three and five years (Fig. 1). Our findings were then examined in two independent cohorts, the ALSPAC and Generation R described below.

Behavioral profiles were based on Tsotsi et al. (2023), who identified latent profiles reflecting primarily internalizing difficulties, primarily aggressive behavior, co-occurring internalizing difficulties and aggression, and low-symptom/normative development. In the present study, we refer to the primarily aggressive profile as the externalizing profile, consistent with this previously derived latent profile structure. We integrated genome-wide association analyses, SNP-based heritability estimation, polygenic score analyses, epigenome-wide association analyses of cord-blood DNA methylation, and weighted co-methylation network analysis. Rather than assuming that each molecular layer would yield equally strong evidence, we asked whether a coherent developmental pattern would emerge across genetic and epigenetic analyses. We hypothesized that molecular signals would be modest in early childhood and stronger by age five than by age three, with polygenic analyses providing the clearest evidence and cord-blood methylation contributing complementary, hypothesis-generating biological context.

## Methods and Materials

### Sample and data processing

#### The Mother, Father, and Child Cohort study (MoBa)

Participants were recruited from all over Norway from 1999 to 2008. The women consented to participation in 41% of pregnancies. The cohort includes approximately 113,632 children, 94,834 mothers and 75,229 fathers ^25^. The present study focuses on child data, with umbilical cord blood samples collected at birth for most participants. This study is based on version 12 of the quality-assured MoBa dataset, released for research in 2021. The establishment of MoBa and initial data collection were approved by the Norwegian Data Protection Agency and the Regional Committees for Medical and Health Research Ethics (REK:185800). The MoBa cohort is currently regulated by the Norwegian Health Registry Act. This study received ethical approval from REK (2009/1899–7; 2013/2061) and the Norwegian Data Inspectorate, with informed consent obtained from all participants prior to participation.

### The Generation R Study

The Generation R Study is a population-based cohort on health and development from fetal life onwards ^27,28^. Pregnant women residing in the study area of Rotterdam, the Netherlands, with an expected delivery date between April 2002 and January 2006 were invited to participate. The study has been approved by the Medical Ethical Committee of Erasmus MC, University Medical Center Rotterdam. Written informed consent was obtained from parents of all children. In total, 9,778 mothers were enrolled in the study and gave birth to 9,749 live-born children. In two subsamples of children of parents born in the Netherlands, the cord blood of 1,393 children was epigenotyped with the Illumina Infinium HumanMethylation450 BeadChip (Illumina Inc. Sand Diego, CA) and the cord blood of another 1,115 children was epigenotyped with the Illumina Infinium MethylationEPIC v1.0 BeadChip. Of these, 1,145 and 897 children had data available on the Child Behavior Checklist ^29^ at age 6 years (CBCL/6–18), respectively. Of these, we analyzed the data of the 1,076 and 843 children had complete data on all covariates, respectively. There were no siblings present within or between these datasets.

#### The ALSPAC study

Pregnant women resident in Avon, UK with expected dates of delivery between 1st April 1991 and 31st December 1992 were invited to take part in the study. The initial number of pregnancies enrolled was 14,541. Of the initial pregnancies, there were 13,988 children who were alive at 1 year of age (Boyd et al., 2013; Fraser et al., 2013). The total sample size for analyses using any data collected after the age of seven is therefore 15,447 pregnancies. Of these 14,901 children were alive at 1 year of age. The study website contains details of all available data through a fully searchable data dictionary and variable search tool (http://www.bristol.ac.uk/alspac/researchers/our-data/). Our study population is drawn from the Accessible Resource for Integrated Epigenomics Studies (ARIES) project, a sub-study drawn from the ALSPAC mother-child cohort on a subset of 1,018 mother-child pairs, which has DNA methylation available. For inclusion, participants were required to have DNA methylation data at birth and Strengths and Difficulties Questionnaire (SDQ) data at age 4, resulting in a final sample size of 706 individuals. Ethical approval was obtained from the ALSPAC Ethics and Law Committee and the Local Research Ethics Committees. Informed consent for the use of all data collected was obtained from participants following the recommendations of the ALSPAC Ethics and Law Committee at the time. Participants can contact the study team at any time to retrospectively withdraw consent for their data to be used. Study participation is voluntary and during all data collection sweeps, information was provided on the intended use of data.

### Measures

#### MoBa

Child behavior was assessed using items from the Child Behavior Checklist (CBCL; Achenbach & Rescorla, 2000), as described in Tsotsi et al. (2023). Mothers rated their children’s behaviors on a three-point scale ranging from 0 (not true) to 2 (very true). In Tsotsi et al. (2023), exploratory and confirmatory factor analyses were first used to identify the internalizing difficulties and aggression indicators at ages three and five years. Internalizing difficulties were assessed with nine items at age three and ten items at age five, with raw scores ranging from 0 to 18 and 0 to 20, respectively. Aggression was assessed with four items at both ages, with raw scores ranging from 0 to 8.

These factor-derived internalizing and aggression scores were then entered into latent profile analyses to identify data-driven behavioral profiles at each age. The analyses identified four profiles: low-symptom/normative, primarily internalizing, primarily aggressive, and co-occurring internalizing difficulties and aggression. In the present study, we refer to the primarily aggressive profile as the externalizing profile, reflecting the externalizing-related manifestation captured by this previously derived profile structure.

Latent profile analysis was performed separately for ages three and five to identify behavioral subgroups. Based on model performance, four groups were identified at each age (Suppl Figs. S1 and S2): a group with low or normal levels of behavioral problems (normative group), a group with primarily internalizing difficulties (internalizing), a group with primarily externalizing behaviors (aggression), and a group with co-occurring internalizing and externalizing behaviors. The distribution of children across these groups at each age is shown in Suppl Fig. S3. Similar Latent profiles were developed in Gen R and ALSPAC.

### The Generation R Study

The Child Behavior Checklist (CBCL/6–18) was reported by the main caregiver at age 6 ^29^. Items were scored on a 3-point Likert scale 3-point Likert scale (0 = not true, 1 = somewhat or sometimes true, and 2 = very true or often true). Weighted sumscores were computed for internalizing behavior (31 items comprising anxious/depressed, withdrawn/depressed and somatic complaints) and aggressive behavior (19 items), allowing for up to 25% missing.

#### ALSPAC

The parent-reported SDQ was administered to children at a median age of four. The SDQ consists of five domains: emotional problems, peer problems, conduct problems, hyperactivity/inattention, and prosocial behavior. Each subscale consists of five items scored on a 3-point Likert-type scale (not true, sometimes true, and certainly true) with a response option for can’t say/not applicable. Items were summed to generate an overall score for each domain that ranges from 0 to 10. In line with SDQ recommendations, we derived an ‘Internalizing problems’ variable by summing emotional problems and peer problems for a composite score. Externalising problems was indexed by conduct problems. A latent class analysis with a three-profile solution was carried out with these variables and the posterior probabilities of belonging to class 3 were used as the co-occurring phenotype used in EWAS analysis.

### Genotypes and Imputation

#### MoBa

The MoBa genetics data is managed by a centralized core team, with strict quality control procedures applied before data sharing across subprojects. The present study is based on version v1 of the dataset, which was generated using a carefully designed pipeline that accounts for the unique family structure in the MoBa study while performing imputation ^30^. This pipeline imputed and released 6,981,748 hard-coded autosomal SNPs for 207,569 unique individuals of European ancestry, along with sex chromosome genotypes for a subset of individuals. The present study did not use sex chromosome data or parental genotypes. Among the children with available behavioral data, up to 40,149 had quality-controlled genotype data and the exact number varies by behavioral and age group (Suppl Fig. S3).

Genotype-based principal components (PCs) were computed following standard procedures. Relatively rare SNPs, defined as those with a minor allele frequency (MAF) below 0.05, and SNPs failing Hardy-Weinberg equilibrium tests (*P* < 10^−6^) were removed. The remaining SNPs were thinned using a sliding window approach to remove highly correlated neighboring SNPs, as implemented in PLINK 2 ^31^ with the parameters *–indep-pairwise* 100 50 0.1. The resulting thinned genotype data were then used as input for principal component analysis (PCA) using the PLINK *–pca* command. The top ten PCs were included in downstream analyses to account for subtle population stratification.

### DNA Methylation

#### MoBa

DNA methylation profiles for MoBa participants were processed and quality-checked by a core team across ten data waves, following the pipeline documented at https://github.com/folkehelseinstituttet/mobagen/wiki/Methylation#met001.Briefly, preprocessing was conducted separately for each wave using the minfi R package^32^, following the 12-step protocol outlined in the pipeline.

The present study includes only child data and excludes waves designed for specific drug effects (waves 006 and 007, focused on Paracetamol) or those that included only ADHD patients. Waves 001 to 003 were based on the Illumina Infinium 450K BeadChip (Illumina, CA), while the remaining waves used the Illumina Infinium EPIC BeadChip (Illumina, CA). The quality-checked data were merged into a single dataset, retaining only CpG sites present on both array types. To mitigate batch effects, both wave indicators and chip type were included as covariates in all downstream analyses (n = 407,705). In addition, CpG sites that showed low variation, std deviation < 0.01, were excluded and after these quality assurance steps about 196,794 CpG sites were analyzed for 6,074 individuals. As with genetic data, the number of unique individuals varies across behavioral groups (Suppl Fig. S3). EWAS results were examined for replication purposes in the Generation R and ALPSAC cohorts.

### The Generation R Study

DNA was extracted from cord blood samples taken at birth. Five-hundred nanograms of DNA per sample underwent bisulfite conversion using the EZ-96 DNA Methylation kit (Shallow) (Zymo Research Corporation, Irvine, USA) and further processed with the Illumina Infinium HumanMethylation450 BeadChip (Illumina Inc., San Diego, USA) or Illumina Infinium MethylationEPIC v1.0 BeadChip. Preparation and normalization of the DNA methylation data was performed following the CPACOR workflow ^33^ using the software package R ^34^. Arrays with observed technical problems such as failed bisulfite conversion, hybridization or extension as well as arrays with a sex mismatch were removed. Arrays with a call rate > 95% per sample were carried forward into normalization. The data were quantile normalized and represented as M-values.

#### ALSPAC

DNA extracted from cord blood was bisulfite converted. Samples were processed with the Illumina Infinium HumanMethylation450 BeadChip (Illumina Inc., San Diego, CA). Quality control and EWAS were performed with the meffil package^35^ on R Version 4.5.2. Samples with mismatched genotypes or sex, incorrect relatedness, low concordance with samples collected at other time points, extreme dye bias, or poor probe detection were excluded prior to normalization. Functional normalization was performed with 10 control probe principal components while including slide as a random effect. Probes were removed if they showed a detection *p*-value > 0.01 or a bead count below 3 in over 10% of samples. A total of 485,512 autosomal CpG sites passed these quality control steps.

### Statistical Analysis

#### Genome-Wide Association Studies (GWAS)

Given the sample size constraints, we employed a case-control design instead of analyzing raw behavioral scores as continuous traits. Case status was defined from the previously established latent profile structure, which enabled data-driven identification of low-symptom/normative, primarily internalizing, primarily aggressive, and co-occurring profiles. These profiles have previously been shown to differentiate depressive, anxiety, conduct, and oppositional defiant symptoms at age eight, supporting their validity as meaningful early childhood risk profiles. We performed GWAS for three behavioral contrasts: internalizing versus low symptom group, aggression versus low, and co-occurring versus normal. Approximately 5% of the included children are siblings. To account for family structure, we used generalized linear mixed-effects models, where family identifiers were included as random intercept effects. Fixed effects included the SNP, the top ten PCs, sex, and genotyping batch indicators. The genetic relationship matrix was computed using the same thinned genotype data as for the PC analysis. GWAS was performed using GLMM models implemented in GCTA v1.94 ^36^ with the command *–fastGWA-mlm-binary*. Genome-wide significance was defined as *P* < 5 × 10^−8^.

#### SNP Heritability

The proportion of phenotypic variance attributable to genome-wide SNPs was estimated using GCTA’s restricted maximum likelihood models. The significance of SNP-heritability estimates was assessed using the likelihood ratio test implemented in GCTA with the *–reml* command. To correct for multiple testing, we applied the Benjamini-Hochberg false discovery rate (FDR), considering FDR ≤ 0.05 as statistically significant.

#### Polygenic Risk Score (PGS) Analysis

To investigate genetic correlations between childhood behavioral problems and major neuropsychiatric conditions, cognitive traits, we conducted a PGS analysis. Summary statistics were obtained from GWAS on schizophrenia (SCZ) ^16^, bipolar disorder (BIP) ^18^, depression (DEP) ^37^, autism spectrum disorder (ASD)^38^, attention deficit hyperactivity disorder (ADHD) ^39^, generalized anxiety disorder (ANX) ^40^, general intelligence (COG) ^41^, educational attainment (EDU) ^42^, neuroticism (NEU) ^43^, and obsessive-compulsive disorder (OCD) ^44^. As a negative control, we used hair color (HC) ^45^. Summary statistics based on European samples were aligned to high-quality SNPs from the HapMap3 dataset before analysis. To account for winner’s curse bias in SNP effect sizes, we applied a Bayesian mixture model, PRS-CS^46^. The posterior-corrected SNP effects were used as allele weights in computing PGS via the PLINK –score function.

We used multiple logistic regression models to assess the effect of PGS on the probability of exhibiting abnormal behavior compared to the normal group. The same covariates used in the GWAS analysis were included in the PGS models. To correct for multiple testing, we applied FDR procedure and results were considered statistically significant at FDR ≤ 0.05.

#### Epigenome-Wide Association Studies (EWAS)

We performed EWAS using the MOMENT model of the OSCA package in MoBa ^47^, which, despite its conservative nature, effectively reduces batch effects by explicitly modeling neighboring methylation sites. Because DNA methylation levels can be either causal or consequential, this study tested whether methylation levels associate with the probability of being in an abnormal behavioral group compared to the normal group. In total, we analyzed 196,794 cytosine-phosphate-guanine (CpG) sites. To further minimize the risk of confounding, we included an extensive set of covariates, including gestational age at birth, maternal smoking during pregnancy (current, former, never), delivery mode (caesarean or vaginal), DNA methylation profiling wave and BeadChip type indicators, estimated immune cell proportions for six immune cell types, CD8 T cell, CD4 T cell, NK cell, monocyte, B cell and granulocyte, as well as DNA methylation based top ten principal components as covariates. We applied a p-value threshold of ≤ 1 × 10^−7^ to identify differentially methylated positions (DMPs).

To replicate significant DMPs identified in MoBa, logistic regression models were applied to the Generation R and ALSPAC DNA methylation data. The co-occurring profilevariable was used as dependent variable, and each of the significant DMPs was used as predictor, along with a set of covariates similar to that used in the MoBa analysis. These include the estimated proportions of the six immune cells, sex of child determined at birth, gestational age at birth, birth mode, maternal smoking during pregnancy. In addition, in the ALSPAC analysis, Surrogate variable analysis (SVA) resulted in 32 surrogate variables and was used to account for unmeasured sources of technical and biological variation.

#### Variance in Behavioral Traits Explained by DNA Methylation

We estimated the proportion of variance in behavioral traits attributable to genome-wide DNA methylation variation using –reml function of the OSCA software. The same set of covariates used in the EWAS analysis was included in these models. Multiple testing correction was performed using the Benjamini-Hochberg FDR procedure, with FDR ≤ 0.05 considered statistically significant.

#### Weighted Correlation Network Analysis (WGCNA)

We performed weighted correlation network analysis (WGCNA) ^48^ to explore the coordinated influence of DNA methylation on childhood behavioral problems. To reduce statistical noise and computational cost, we further restricted this analysis to CpGs that have a minimal standard deviation > 0.05, and medium absolute deviation > the 75% quantile. This reduced the number of CpGs from 196,794 down to 37,499.

We used the *pickSoftThreshold* function in the WGCNA package to determine the optimal power parameter for the scale-free network assumption, scanning values from 1 to 12. We decided the optimal power value to 2, which has an *SFT.R.sq* value of 0.937 and a *mean.k* 3.16. Based on this selected power, the *blockwiseModules* function was used to derive DNA methylation modules (MEs), with the following settings: correlation *type*=bicor, *minModuleSize* = 300, *TOMType*=’unsigned’, *deepSplit* = 1, *maxBlockSize* = 3000, *mergeCutHeight* = 0.5. Other parameters were set to the default values. To summarize the functional relevance of these modules, eigengenes (the first principal component of the co-methylation networks) were computed using *moduleEigengenes*.

The association between eigengenes and behavioral groups was assessed using logistic regression, adjusting for the same covariates used in EWAS to control for confounding.

#### Module Pathway Enrichment Analysis

We performed the pathway enrichment analysis for CpGs included in the DNA methylation modules, ME1 and ME3, using the standard package of in the field, missMethyl ^49^. Pathways in both the KEGG ^50^ and GO BP databases (geneontology.org) were tested for enrichment. Since many gene sets within each database share genes and are therefore correlated, we applied the FDR procedure to correct for multiple testing, considering FDR ≤ 0.05 as the significance threshold.

#### DNA Methylation Quantitative Trait Locus (mQTL) Analysis

For each identified DMP, we conducted cis-mQTL analysis using GCTA with the –fastGWA-mlm command. SNPs located within a 1 Mb radius of a DMP were analyzed. The same covariates used in the GWAS analysis were included in the models to control for confounding effects. To correct for multiple testing, we applied the Bonferroni correction.

## Results

### Study overview

Using the MoBa cohort we examined internalizing, aggression, and co-occurring behavioral profiles at ages three and five years. Behavioral profiles were defined by latent profile analysis of CBCL ^29^ measures at each age, yielding typical, internalizing, aggression, and co-occurring groups. The distribution of children across these groups at each age is shown in Supplementary Figs. S1 and S2. We then investigated genetic and epigenetic correlates of the three non-normative profiles relative to the normative group at each age (Fig. 1).

### Genetic contribution to Child Behavior Problems

Genome-wide SNP-based heritability (h^2^_SNP_) estimates were modest overall. At age three, internalizing and aggression profiles showed significant estimates (h^2^_SNP_ = 0.046, 0.061; standard error [*se*] = 0.013, 0.013; *FDR* = 5.26 × 10^−4^, 6.06 × 10^−6^; respectively), whereas co-occurring problem did not survive multiple-testing correction. By age five, all three behavioral phenotypes showed significant estimates h^2^_SNP_, with similar effect magnitudes: internalizing h^2^_SNP_ = 0.033 (*se* = 0.013, *FDR* = 1.151 × 10^− 3^), aggression h^2^_SNP_ = 0.037 (*se* = 0.014, *FDR* = 3.791 × 10^− 3^), and co-occurring h^2^_SNP_ = 0.035 (*se* = 0.015, *FDR* = 9.499 × 10^− 3^). These findings indicate that common variant contributions are detectable but modest and become more consistently evident at age five.

### Genetic Variants Associated with Child Behavioral Problems

Across the six GWAS performed, only co-occurring problems at age 5 yielded a genome-wide significant locus (Suppl. Figs. S4-S7). The lead variant, rs73161798, mapped to a gene-dense region on chromosome 13q12, with effect allele A showing log(OR) = 1.08 (*se* = 0.19, *P* = 3.26 × 10^− 8^) (Fig. 2A). Several genes with potential relevance to neurodevelopmental or behavioral biology were located in this locus, for example, the general transcription factor IIIA gene (*GTF3A*), the ubiquitin-specific peptidase 12 (*USP12)*, and the Ras-like protein family member 11A (*RASL11A*). No comparable signal was observed for the other behavioral profiles or age groups. Genomic inflation was minimal (lambda = 1.002–1.012; Suppl. Figs S5 and S7), supporting the robustness of the association.

#### Polygenic score associations

Polygenic score analyses showed broader and more coherent associations at age five than at age three (Fig. 2B; see Suppl Figs. S9, S10, Tables S1 and S2 for details). Among psychiatric traits, neuroticism PGS was associated with nearly all behavioral profiles across ages (FDR adjusted *P* < 0.05), while educational attainment PGS generally showed inverse associations with behavioral risk. Internalizing problems at age five displayed the broadest pattern, being associated with all eight psychiatric PGSs, compared with only three at age three. One notable age-dependent pattern was observed for ADHD PGS: it was positively associated with aggression at age three (*OR* = 1.09, 95%CI: 1.05–1.134, *FDR* = 1.14 × 10^− 4^) but with internalizing at age five (*OR* = 1.21, 95%CI:1.157–1.266, *FDR* = 2.22 × 10^− 15^), and negatively associated with aggression at age five (*OR* = 0.92, 95%CI:0.881–0.965, *FDR* = 2.15 × 10^− 3^). Overall, these findings suggest that polygenic liability is developmentally structured and more detectable at age five.

### Longitudinal pattern across ages

Among children with behavioral data available at both ages, summary analyses showed that stable internalizing profiles carried the broadest polygenic burden across cognitive, neurodevelopmental, and psychiatric score domains (Fig. 2C). Stable aggression and co-occurring profiles also showed polygenic signal, but with less consistent cross-domain loading. These results support the view that persistence across early childhood may index a more structured genetic liability.

### DNA Methylation and childhood Behavioral Problems

We estimated the proportion of behavioral variation explained by epigenome-wide DNA methylation in umbilical cord blood. Only co-occurring problems at age five showed significant DNA methylation-associated variance (variance explained = 0.297, *se* = 0.071). These findings suggest that emergent co-occurring behavioral problems may carry a measurable epigenetic signal, whereas other behavioral profiles appear to be more captured by genotype-based analysis. Full estimates for all traits are provided in Supplementary Tables S3 and S4.

### Single-site methylation associations are limited but support a developmental contrast

After quality control of the Illumina 450 and EPIC arrays and removal of sites that failed quality control or showed little variation, we performed genome-wide differential methylation analysis (EWAS) on 196,794 sites (Methods) in MoBa. At age three, cg10976481, annotated to the first exon of the Tankyrase gene (*TNKS*), was associated with the co-occurring problems (beta = −0.024, se = 0.004, *P* = 9.03 × 10^− 8^) (Fig. 3A). At age five, we identified three independent differentially methylated sites (Fig. 3B-D): cg18224993 in the enhancer region of the Teneurin transmembrane protein 4 (*TENM4*, previously named ODZ4; beta = −0.03, *se* = 0.003, *P* = 6.38 × 10^− 8^), cg03585974 near the transmembrane protein 239 (*TMEM239*; beta = −0.027, *se* = 0.005, *P* = 7.31×10^− 9^), and the intergenic site cg03106395 (beta = −0.028, *se* = 0.005, *P* = 2.21 × 10^− 8^). We did not identify significant sites for internalizing or aggression symptom profiles. Inflation statistics did not indicate substantial bias due to cryptic relatedness nor unmodelled confounding (lambda: 0.82–0.98; Suppl Figs. S12–16), and the volcano plots showed no obvious abnormalities.

We next examined these four differentially methylated sites in two external cohorts, the Generation R and the ALSPAC datasets (Methods). In Generation R, cg18224993 in the *TENM4* locus showed consistent direction of effect and a nominal association with the co-occurring problems (z-score = −2.053, *P* = 0.04) with the Illumina EPIC array data (n = 843). In the Illumina 450K data (n = 1,145) this probe showed consistent effect direction but not significant (*P* > 0.05). In ALSPAC, none of the four sites showed nominal evidence of associations (all *P* > 0.05; n = 706). Taken together, these external lookups provide partial support for the *TENM4* signal in Generation R, while indicating that the single-site methylation findings should be interpreted cautiously and require further evaluation in larger harmonized samples.

Overall, the single-site methylation findings were modest and focal rather than widespread (Fig. 3E). The age-five co-occurring profile showed the clearest methylation signal, including a CpG in the *TENM4* locus with partial external support in Generation R but not ALSPAC. As in the genetic analyses above, the contrast between ages three and five was notable: methylation signals were weaker and scattered at age three, but more structured and biologically interpretable at age five.

### Co-methylation network analysis identifies biologically coherent modules at age five

Because single-site methylation results were limited, we asked whether coordinated methylation structure could be detected at the network level. Weighted co-methylation network analysis identified five eigengene modules (ME1–5, Fig. 4A). Correlations among the eigengenes suggested three separable module groups, with ME1 and ME2 negatively correlated (r = −0.35), ME4 and ME5 positively correlated (r = 0.43), and ME3 itself as a unique group, indicating that these groups may capture distinct biological processes. Using logistic regression while controlling relevant covariates, we found that at age five, ME1 was associated with co-occurring problems (*P* = 0.045) and ME3 with aggression (*P* = 3.73 × 10^− 3^). In contrast, age-three module associations were weaker and less specific (see Suppl. Table S5 for full results).

Although pathway enrichment for the methylation modules did not survive multiple-testing, nominally enriched pathways were biologically coherent (Fig. 4C; Suppl. Tables S6-S9). For ME1, selected terms converged on neurodevelopmental and synaptic themes, including neuron development, dendrite development, neuron projection morphogenesis, synapse organization, axon guidance, glutamatergic synapse, and related signaling pathways. For ME3, nominal enrichment highlighted pathways related to neuronal plasticity and signaling, including collateral sprouting, presynaptic membrane regulation, GABAergic synapse, and lipid- or signaling-related processes.

These results do not provide definitive pathway-level evidence. Rather, they offer hypothesis-generating biological context that is consistent with the broader developmental pattern observed across the study

## Discussion

In this multi-layer molecular analysis of early childhood behavioral profiles in MoBa, we found that molecular correlates of internalizing, aggressive, and co-occurring problems were modest overall but become more coherent by age five. The strongest and most consistent evidence came from polygenic score analyses, which showed broader associations at age five than at age three across psychiatric, cognitive, and neurodevelopmental domains. GWAS identified one genome-wide significant locus for co-occurring problems at age five, whereas single-site cord-blood methylation associations were limited. However, at age five co-methylation network analysis identified modules associated with co-occurring and aggression profiles, with nominal enrichment for neurodevelopmental, synaptic, and neuronal-plasticity themes. Together, these findings suggest that molecular correlates of early behavioral vulnerability are detectable but modest and become more coherent from age three to five.

We identified a genome-wide significant association at chr13q12 for co-occurring problems at age five. The region contains several biologically plausible nearby genes, including *GTF3A*, *USP12*, and *RASL11A*, but the present data do not permit prioritization of a single causal gene. *GTF3A* has been implicated in cellular responses relevant to viral defense in the brain ^51^. USP12 has been linked to adult risk-taking behavior ^20^, and *RASL11A* has been discussed in relation to neurodevelopmental processes ^52^. These annotations provide biological plausibility, but the association cannot yet be assigned to a specific causal gene. The locus therefore requires replication in independent cohorts with comparable early-childhood behavioral profiling, followed by functional annotation to clarify the underlying gene-regulatory mechanism.

The polygenic score analyses provided the clearest evidence that early behavioral profiles index broader psychiatric and neurodevelopmental liability. Internalizing problems at age five showed the broadest pattern of association across psychiatric PGSs, consistent with prior evidence for shared genetic architecture across depression, anxiety, neuroticism, and related internalizing traits ^53^. The age-dependent ADHD PGS pattern was particularly notable. Higher ADHD PGS was associated with aggression at age three, but by age five it was positively associated with internalizing problems and inversely associated with aggression. One possible interpretation is that genetic liability related to ADHD may manifest differently across early development: at younger ages it may be expressed primarily through impulsivity, poor inhibitory control, or dysregulated behavior, whereas at older ages it may increasingly be reflected in emotional dysregulation, anxiety-related behavior, or broader internalizing vulnerability. This interpretation is consistent with evidence that early externalizing, such as aggression, and internalizing symptoms are dynamically related across childhood rather than developmentally fixed ^54–56^. More generally, these findings suggest that the behavioral expression of polygenic liability may shift with age, underscoring the importance of repeated developmental assessment ^57^. Alternatively, changing associations may, at least partly, reflect developmental differences in symptom measurement or phenotypic composition rather than changes in the expression of genetic liability.

Cord-blood DNA methylation contributed a complementary but more exploratory layer of evidence. Because methylation was measured at birth, these signals preceded the behavioral assessments and may reflect prenatal biological variation. However, cord blood is not brain tissue, and these findings cannot establish a direct causal mechanism linking prenatal exposures to later behavior. The sparse single-site EWAS results are therefore best interpreted as focal molecular correlates rather than evidence for a broad methylation architecture of early behavioral problems. The age-five *TENM4*-associated CpG is notable because teneurin-family proteins are conserved cell-adhesion molecules with established roles in axon guidance, synapse formation, neuronal recognition, and circuit wiring ^58,59^, while *TENM4*/*ODZ4* has also been implicated in psychiatric genetics ^60^. Nevertheless, the lack of replication in ALSPAC and Gen R underscores the need for larger samples and tissue-aware functional annotation before strong mechanistic conclusions can be drawn. The co-methylation network results suggest that coordinated methylation patterns may provide biological context beyond single CpG associations. ME1, associated with age-five co-occurring problems, showed nominal enrichment for neurodevelopmental and synaptic pathways, including neuron development, dendrite development, axon guidance, glutamatergic synapse, and long-term potentiation. ME3, associated with age-five aggression, highlighted more mixed but still biologically plausible themes, including GABAergic synapse, presynaptic membrane regulation, cytoskeletal organization, cerebellar development, cholesterol metabolism, and xenobiotic response. Because these enrichments did not survive multiple-testing correction, they should not be interpreted as definitive pathway discoveries. Instead, they support a hypothesis-generating model in which age-five behavioral profiles are accompanied by coordinated cord-blood methylation variation related to neurodevelopmental and neuronal-signaling processes.

Several aspects strengthen this study. First, we used a large, population-based birth cohort with behavioral assessments at two early developmental stages, enabling us to compare molecular correlates at ages three and five. Second, the same broad behavioral domains were available across ages, allowing us to evaluate whether molecular signals became more structured across early childhood. Third, the integration of GWAS, SNP-based heritability, polygenic scores, EWAS, and co-methylation network analysis allowed us to compare multiple molecular layers rather than relying on a single analytic framework. Finally, the use of cord-blood methylation provided a temporally informative measure collected before the emergence of the behavioral profiles studied here.

Several limitations should also be noted. First, behavioral profiles were derived from categorical latent profile assignments, which may simplify the dimensional and probabilistic nature of early behavioral variation ^10^. Although this approach allowed us to contrast co-occurring and domain-specific profiles against a normative group, uncertainty in profile assignment was not fully propagated into the molecular analyses ^61^. Second, behavioral profiles also relied on maternal report and may therefore partly reflect reporter-specific variance. Third, despite the large cohort setting, the number of children in non-normative behavioral groups was more limited than the full MoBa sample, reducing power for GWAS, EWAS, and subgroup-specific analyses. Third, the chr13q12 GWAS locus and methylation findings require independent replication with large samples. Fourth, cord-blood methylation is an accessible early-life tissue but does not directly index methylation patterns in the developing brain. Finally, pathway enrichment results were nominal and should be interpreted as hypothesis-generating rather than confirmatory.

In conclusion, this study supports a developmental, multi-layer view of early childhood behavioral vulnerability (Fig. 5). Co-occurring behavioral problems were not characterized by a single dominant molecular signature, but by modest signals distributed across genetic and epigenetic layers, most clearly detectable by age five. Polygenic score analyses provided the strongest evidence for shared psychiatric, cognitive, and neurodevelopmental liability, whereas cord-blood methylation contributed complementary but more exploratory biological context through sparse CpG findings and network-level structure. These results highlight the value of integrating behavioral profiling with genomic and epigenomic data in early childhood, while underscoring the need for replication, longer follow-up, and tissue-aware interpretation of methylation findings.

## Supplementary Material

Supplementary Files

This is a list of supplementary files associated with this preprint. Click to download.

• SupplTablesBekkhus.xls

• SupplfiguresBekkhus.pdf

## Figures and Tables

**Figure 1:**
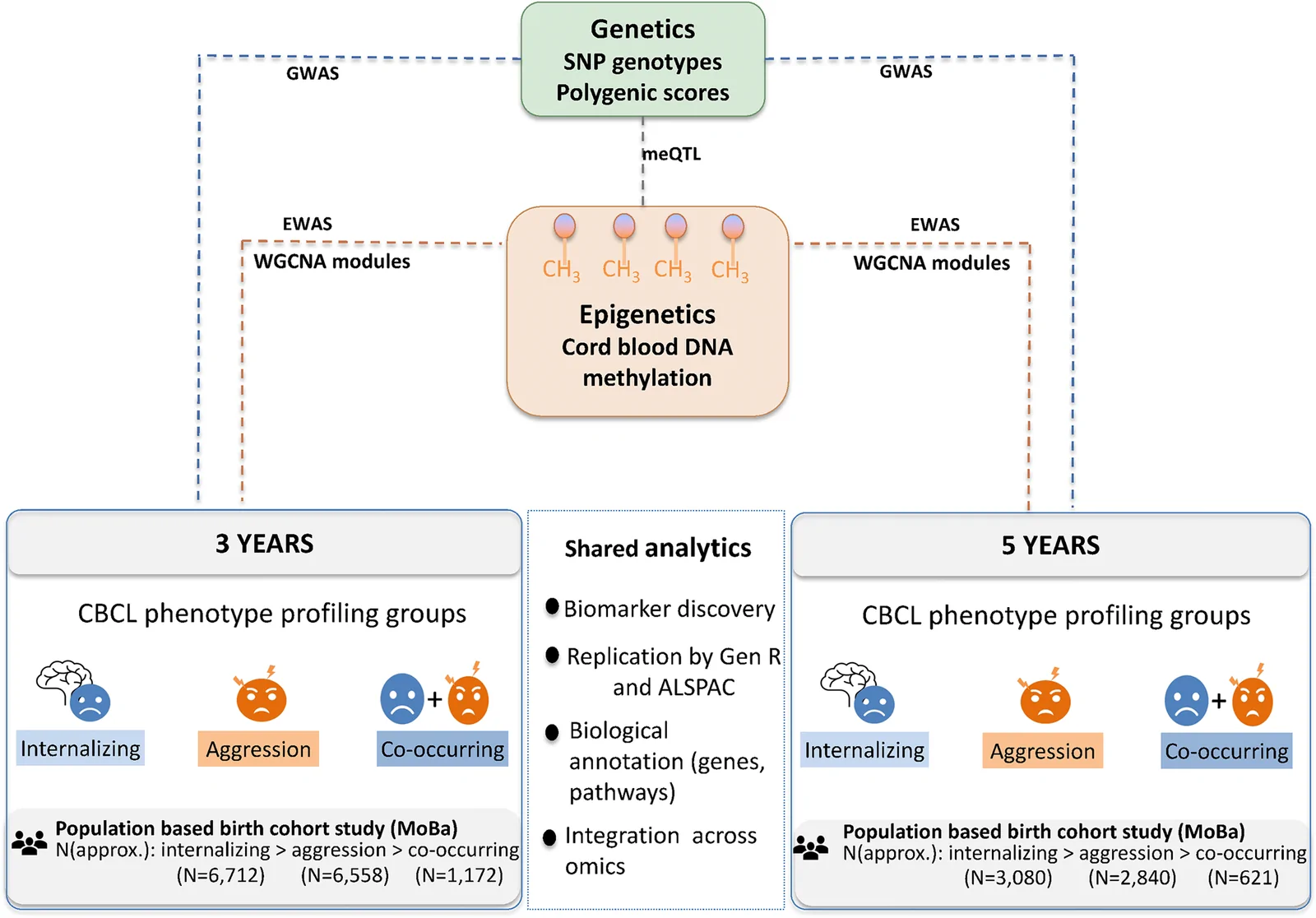
Study design and multi-layer molecular analysis of early childhood behavioral profiles. Internalizing, aggression, and co-occurring behavioral profiles were defined using Child Behavior Checklist (CBCL)-based phenotype profiling at ages three and five years in the Norwegian Mother, Father and Child Cohort Study (MoBa). At age three, the approximate sample sizes were 6,712 for internalizing, 6,558 for aggression, and 1,172 for co-occurring problems. At age five, the approximate sample sizes were 3,080 for internalizing, 2,840 for aggression, and 621 for co-occurring problems. Genetic analyses included SNP genotypes, genome-wide association analysis (GWAS), and polygenic score analyses. Epigenetic analyses used cord-blood DNA methylation and included epigenome-wide association analysis (EWAS), methylation quantitative trait locus (meQTL) information, and weighted co-methylation network analysis (WGCNA). Shared downstream analyses included biomarker discovery, biological annotation of genes and pathways, and integration across molecular layers.

**Figure 2:**
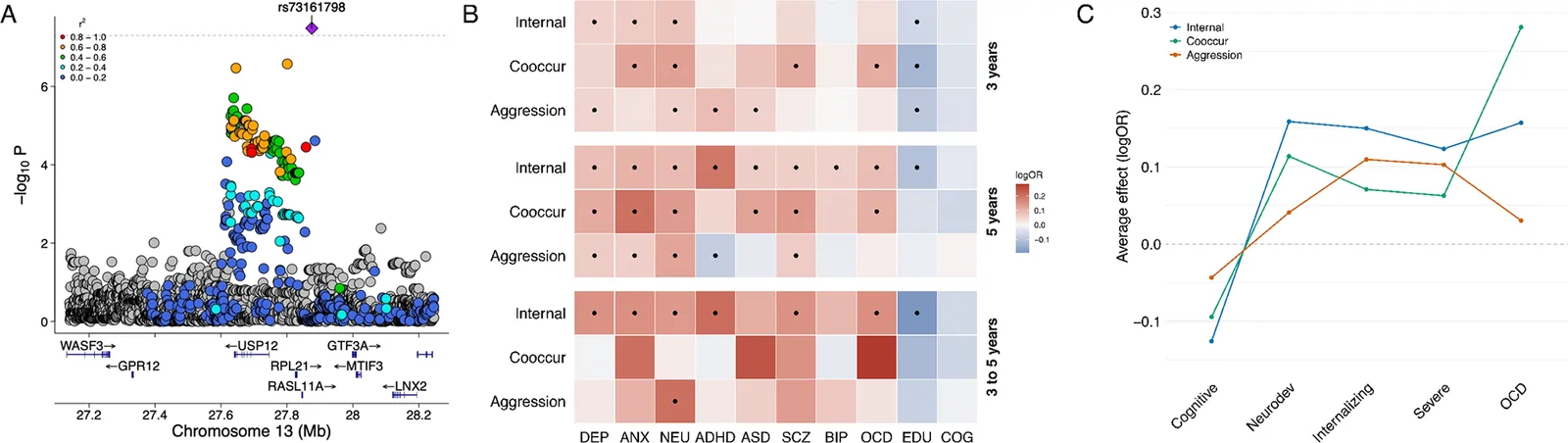
Genetic findings for childhood behavioral profiles. (**A**) Regional association plot for the genome-wide significant locus associated with co-occurring problems at age five. The lead variant, rs73161798, maps to chromosome 13q12, with nearby genes including WASF3, GPR12, USP12, RPL21, RASL11A, GTF3A, MTIF3, and LNX2. Points are colored according to linkage disequilibrium with the lead variant. The dashed horizontal line indicates the genome-wide significance threshold. (**B**) Polygenic score associations across behavioral profiles and ages. Heatmap colors represent log odds ratios for associations between polygenic scores and internalizing, co-occurring, and aggression profiles at age three, age five, and persistent or transitional profiles from age three to age five. Black dots indicate associations passing multiple-testing correction. Polygenic scores include depression (DEP), anxiety (ANX), neuroticism (NEU), attention-deficit/hyperactivity disorder (ADHD), autism spectrum disorder (ASD), schizophrenia (SCZ), bipolar disorder (BIP), obsessive-compulsive disorder (OCD), educational attainment (EDU), and cognitive performance (COG). (**C**) Average polygenic score effects across broader score domains for longitudinal behavioral profiles. Values represent average log odds ratios across cognitive, neurodevelopmental, internalizing, severe psychiatric, and obsessive-compulsive disorder-related polygenic score domains.

**Figure 3:**
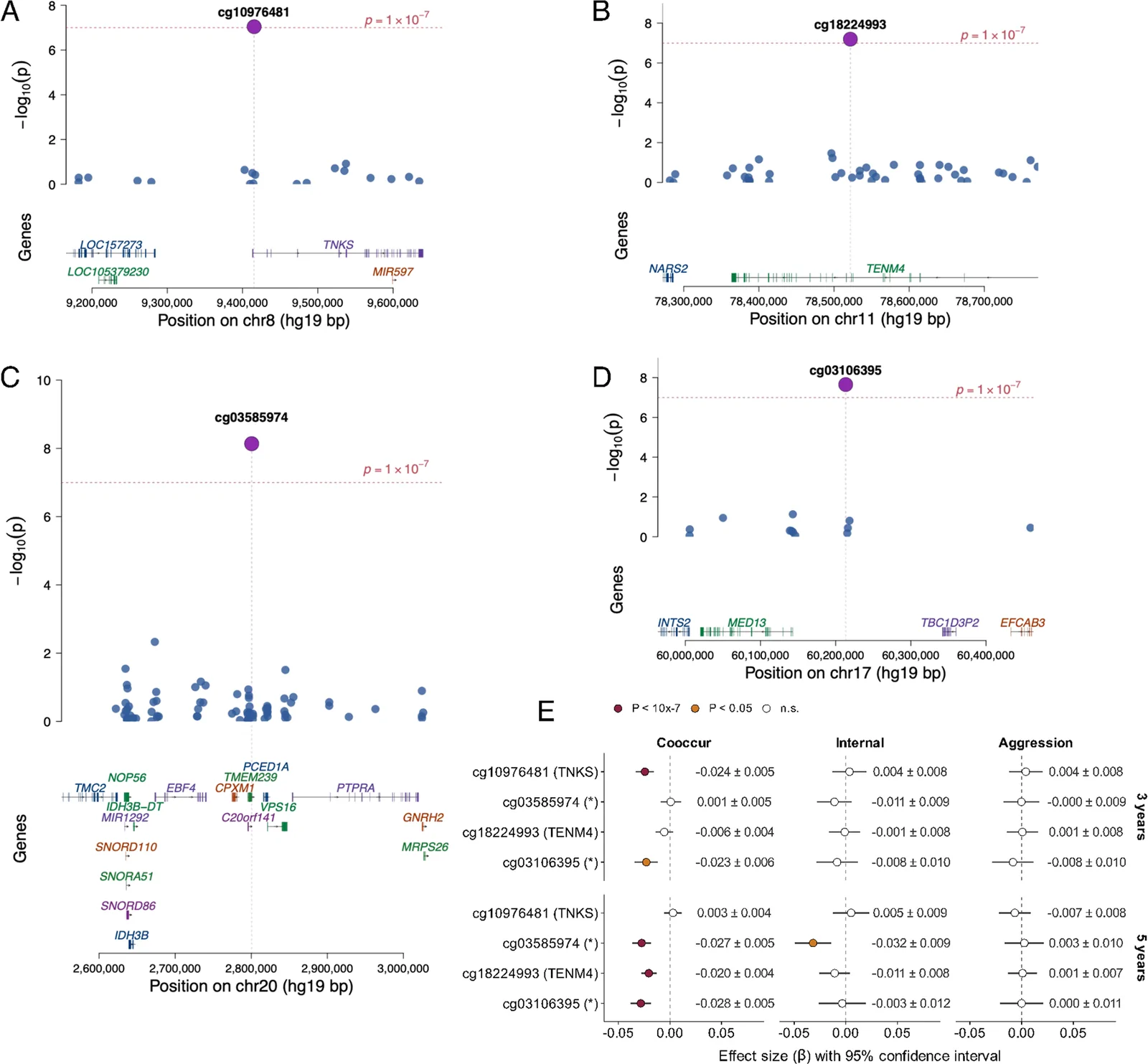
Single-site cord-blood DNA methylation associations with childhood behavioral profiles. (**A**) Regional methylation association plot for cg10976481, annotated to *TNKS*, associated with co-occurring problems at age three. (**B–D**) Regional methylation association plots for age-five co-occurring problems, showing cg18224993 in the *TENM4* locus, cg03585974 near *TMEM239*, and the intergenic CpG cg03106395, respectively. The dashed horizontal line indicates the epigenome-wide significance threshold. (**E**) Effect-size summary for the four differentially methylated CpGs across behavioral profiles and ages. Points show regression coefficients and horizontal lines show 95% confidence intervals for co-occurring, internalizing, and aggression profiles at ages three and five. Filled points indicate epigenome-wide significant associations, colored points indicate nominal associations, and open points indicate non-significant associations.

**Figure 4:**
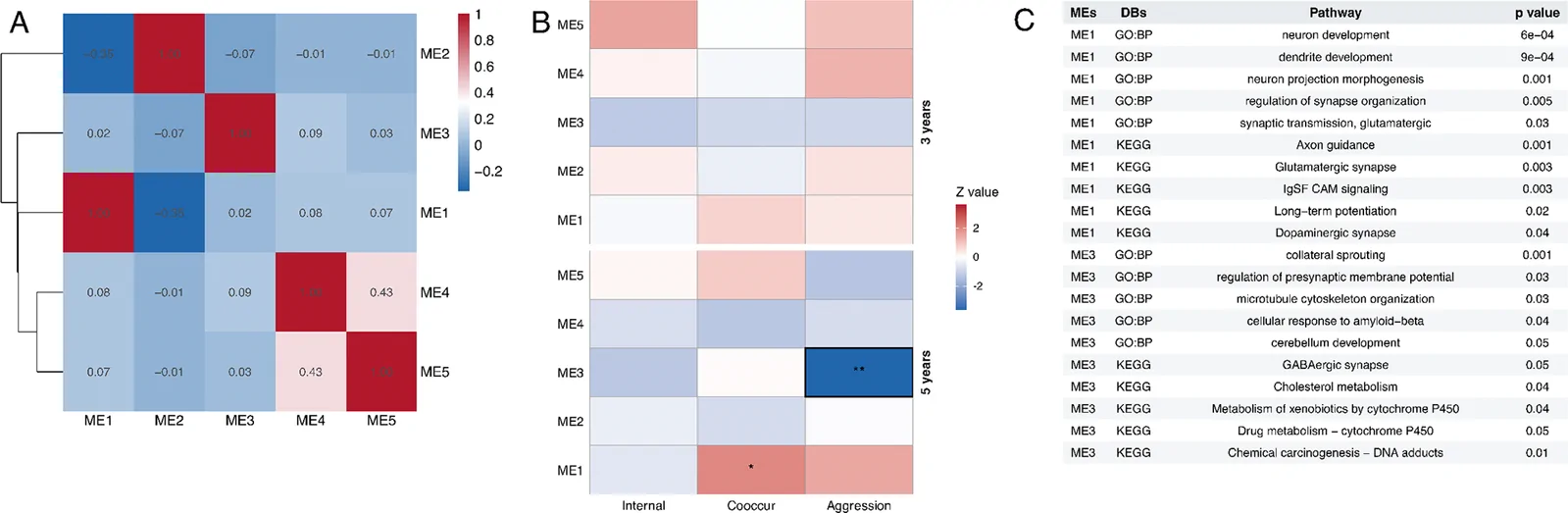
Co-methylation network analysis of cord-blood DNA methylation. (**A**) Correlation heatmap of weighted co-methylation network analysis module eigengenes. Five modules were identified. ME1 and ME2 showed a negative eigengene correlation, whereas ME4 and ME5 showed a positive eigengene correlation, suggesting partially separable axes of methylation variation. (**B**) Associations between methylation module eigengenes and behavioral profiles at ages three and five. Heatmap colors represent regression Z values from logistic regression models adjusted for relevant covariates. Rows correspond to module eigengenes and columns to internalizing, co-occurring, and aggression profiles. Asterisks indicate nominally significant module–phenotype associations. (**C**) Selected nominally enriched pathways for modules associated with age-five behavioral profiles. The table shows biologically prioritized Gene Ontology Biological Process and KEGG pathways for ME1 and ME3, together with nominal enrichment P values. Pathways were selected to summarize neurodevelopmental, synaptic, neuronal-signaling, cytoskeletal, lipid-metabolic, and xenobiotic-response themes. These enrichments did not survive multiple-testing correction and should be interpreted as hypothesis-generating.

**Figure 5:**
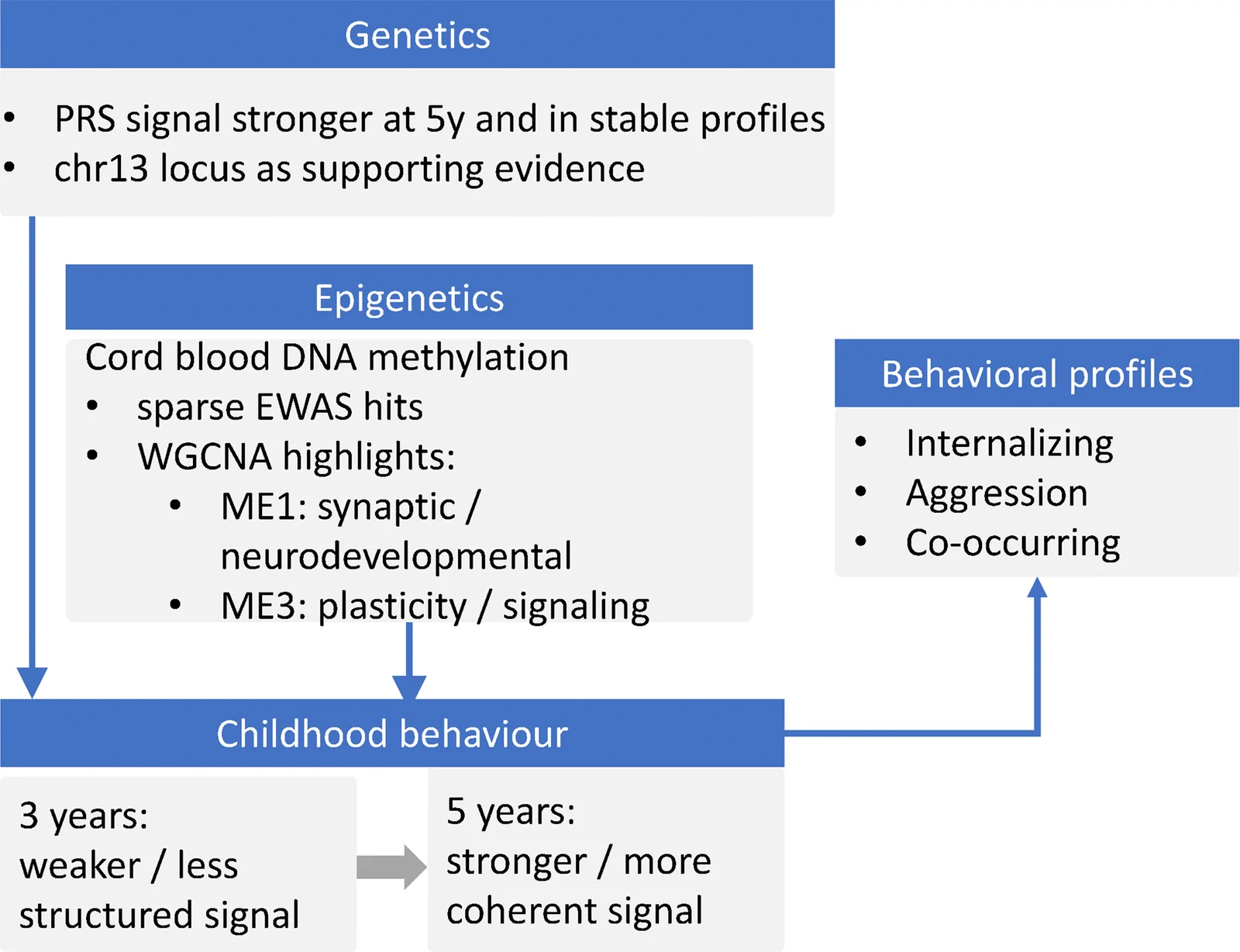
Conceptual model summarizing developmental genetic and epigenetic correlates of early childhood behavioral profiles. The integrated model summarizes the main findings across molecular layers. Genetic analyses showed stronger polygenic score signals at age five and in stable behavioral profiles, with the chromosome 13q12 GWAS locus providing supporting locus-level evidence. Cord-blood DNA methylation analyses identified sparse single-site EWAS associations, whereas WGCNA highlighted methylation modules with nominally enriched neurodevelopmental, synaptic, plasticity-related, and signaling-related pathways. Across molecular layers, behavioral profiles at age three showed weaker and less structured signal, whereas age-five profiles showed stronger and more coherent molecular correlates. The model emphasizes internalizing, aggression, and co-occurring profiles as developmentally structured behavioral phenotypes rather than fixed diagnostic categories.

## Data Availability

Genome-wide summary statistics for child behavior problems are available at http://figshare/XXXX. GWAS summary statistics for psychiatric disorders (SCZ, BIP, ASD, ADHD, PTSD, DEP, ANX, and OCD) can be downloaded from https://pgc.unc.edu/for-researchers/download-results/ and for IQ and neuroticism from https://cncr.nl/research/summary_statistics/. Data on educational attainment can be found at http://www.thessgac.org/.

## Code availability

Genome-wide association analysis for child behavior problems was performed using GCTA v1.94 (https://yanglab.westlake.edu.cn/software/gcta/#fastGWA). Polygenic score analysis was conducted with PRS-CS (https://github.com/getian107/PRScs) and PLINK 2.0 (https://www.cog-genomics.org/plink/2.0/). EWAS analysis was performed using the OSCA toolset (https://yanglab.westlake.edu.cn/software/osca). Gene set analysis used the WGCNA R package (https://cran.r-project.org/web/packages/WGCNA/index.html).
